# Comparison of Live and Remote Video Ratings of the Scale for Assessment and Rating of Ataxia

**DOI:** 10.1002/mdc3.13843

**Published:** 2023-08-07

**Authors:** Arian Taheri Amin, Jennifer Faber, Demet Önder, Okka Kimmich, Matthis Synofzik, Tetsuo Ashizawa, Thomas Klockgether, Marcus Grobe‐Einsler

**Affiliations:** ^1^ German Center for Neurodegenerative Diseases (DZNE) Bonn Germany; ^2^ Department of Neurology University Hospital Bonn Bonn Germany; ^3^ Division Translational Genomics of Neurodegenerative Diseases Hertie‐Institute for Clinical Brain Research and Center of Neurology, University of Tübingen Tübingen Germany; ^4^ German Center for Neurodegenerative Diseases (DZNE) Tübingen Germany; ^5^ Houston Methodist Research Institute and Department of Neurology, Houston Methodist Neurological Institute Houston Texas USA

**Keywords:** ataxia, scale for the assessment and rating of ataxia, SARA, remote assessment, teleneurology

## Abstract

**Background:**

Video recordings of neurological examinations are often used in clinical trials. The Scale for Assessment and Rating of Ataxia (SARA) is a widely used clinical scale for ataxic patients. Despite several advantages of video ratings, correlation between live ratings and remote video‐ratings has not been systematically investigated.

**Objective:**

To compare live and remote video assessment of SARA.

**Methods:**

Full SARA examinations of 69 patients with cerebellar ataxia were recorded on video. Live rating from site investigators were compared with remote video rating of three experienced ataxia clinicians using Bland–Altman analysis.

**Results:**

Live and remote video ratings showed a high level of agreement for the complete score (bias = 0.09, with standard deviation = 2.00) and all single SARA items (bias <0.20 for all items).

**Conclusion:**

Remote video ratings of SARA are a reliable means to assess severity of ataxia.

Clinical scales provide an insight into the current state of health or disease severity under predefined conditions. They allow assessment of disease severity, which is essential for patient management and clinical trials. Traditionally, most clinical scales are simultaneously applied and interpreted by an investigator who is performing the examination. The Scale for Assessment and Rating of Ataxia (SARA) is a widely used clinical rating scale for ataxic patients.^1^ It consists of eight items (1: gait, 2: stance, 3: sitting, 4: speech, 5: nose‐finger test, 6: finger chase, 7: fast alternating hand movements, and 8: heel‐shin slide). The SARA sum score ranges from 0 (no ataxia) to 40 points (most severe ataxia). Like all rating scales, SARA is subject to inter‐ and intra‐rater variability and its use in clinical trials is limited by the availability of an examiner.

Recently, video recordings of neurologic examinations and clinical scales have become a frequent means for patient care, rater‐training, and centralized ratings of different movement disorders scales.^2^, ^3^, ^4^, ^5^ In analogy, remote video assessment of SARA examinations are increasingly used to counteract inter‐rater variability (centralized ratings), for training purposes and to assess ataxia severity at patient's home.^6^, ^7^ Contact restrictions during the corona pandemic, but also the mobility and travel impairments inherent in patients with movement disorders, stress the need for remote assessments. Despite the advantages of video ratings, differences between live ratings and remote video‐ratings have not been systematically investigated for SARA.

## Methods

Complete SARA assessments were recorded according to a standardized protocol using a Canon VIXIA HF G21 camera (60 fps). For detailed description of the video protocol, see Figure S1. Participants were enrolled during consecutive study visits of ongoing observational trials within the German Center for Neurodegenerative Diseases (DZNE) in Bonn, Germany. Inclusion criteria were (1) progressive cerebellar syndrome (defined by at least two of gait ataxia, limb ataxia, dysarthria, or oculomotor features) and (2) participation in an observational study of the DZNE (Consortium “Sporadic Degenerative Ataxia with Adult Onset,” European Spinocerebellar Ataxia Type 3/Machado‐Joseph Disease Initiative, Spinocerebellar Ataxia‐Registry, European Friedreich's Ataxia Consortium for Translational Studies, autosomal recessive cerebellar ataxias). The local ethics committee approved the study (IRB: 212/19), and all patients gave informed consent for participation, explicitly including video recordings of neurological examinations.

For all available SARA videos, consensus rating of three clinicians with specialization in ataxia (M.G.E., M.S., and T.K.) was obtained in joint sessions, in addition to the site investigator's live rating. The consensus raters viewed all videos simultaneously via wireless access in teleconference sessions (Lifesize®, Austin, TX, USA). Raters then discussed and agreed on a rating for all items. The consensus raters were not aware of the live ratings from the study visit, and the live raters did not use the video material for their ratings. All available recordings were included in this study. To compare live and video ratings we performed Bland–Altman analysis on the item level and total scores. Bland–Altman plots analyze the deviation of two measurement methods, and a high level of agreement or low measurement difference between two measurement methods is expressed as a low number. The bias indicates whether the video rating over‐ or underestimates disease severity (on the SARA scale) compared to the live rating. Values should be as close to zero as possible and should be <1.0 in this context. The 95% limits of agreement indicate the dispersion of the differences between the rating methods and should cover at least 95% of the plotted data‐points. Additionally, intraclass correlation coefficients (ICC) were calculated to explore test–retest reliability. Calculation of ICC was based on a model with only random effect. Coefficients >0.80 are considered acceptable. All analyses were performed using Graph Pad Prism Version 9.2.0 (Graph Pad®, Boston, MA, USA) and IBM SPSS Statistics 29 (IBM®, Armonk, NY, USA).

## Results

A total of 69 SARA examinations were analyzed live and via remote video ratings. Patient characteristics are given in Table 1. Mean SARA score from live ratings was 15 with a range between 0 and 34. Mean SARA scores from consensus video ratings was 15 with a range between 0 and 33. Distribution of all live and video rating is shown in Figure S2.

**TABLE 1:** Patient characterization

	Validation study (n = 69)
SCA 1 (n)	3
SCA 2 (n)	2
SCA 3 (n)	14
SCA 6 (n)	9
MSA‐C (n)	15
Friedreich ataxia (n)	4
Other recessive ataxia (n)	10
Others (n)	12
Age (years), mean ± SD (range)	51.8 ± 13.9
SARA, mean ± SD (range)	15.2 ± 7.0

*Note*: Bias = 0.09 points, with SD of 2.01 points. >95% of all data points lie within the 95% limits of agreement (−3.85; 4.03).

Abbreviations: SCA, spinocerebellar ataxia; MSA‐C, multiple system atrophy‐cerebellar subtype; SD, standard deviation; SARA, Scale for Assessment and Rating of Ataxia.

**FIG. 1:**
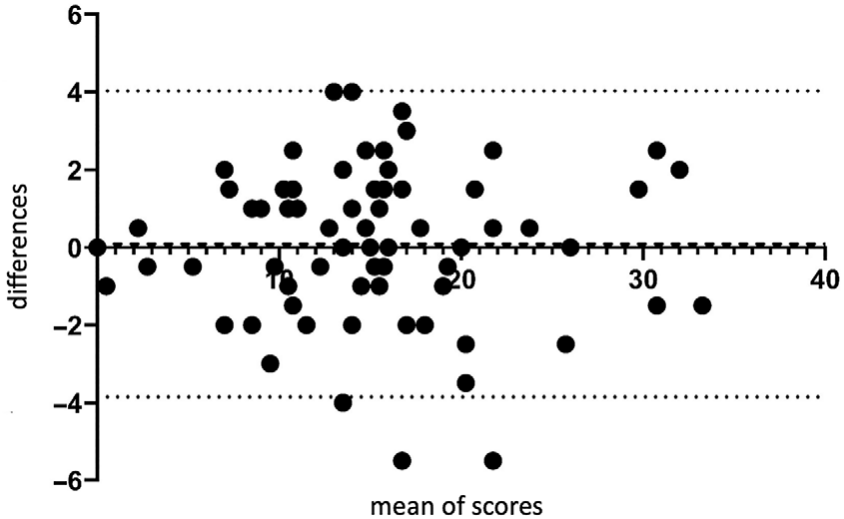
Bland–Altman‐analysis of live versus remote video ratings for the complete Scale for Assessment and Rating of Ataxia score.

Bland–Altman analysis of live and remote video ratings showed high levels of agreement for complete SARA scores (bias = 0.09 with standard deviation [SD] = 2.00 and 95% limit of agreement (95% LA) from −3.85 to 4.03) (Figure 1) and single item analysis (Figure S3). The highest level of agreement was found for stance and heel‐shin slide, followed by fast alternating hand movements. In detail, the bias for the single items was calculated as 0.14 (SD = 0.60, 95% LA = −1.03 to 1.32) for gait, 0.00 (SD = 0.51, 95% LA = −1.01 to 1.01) for stance, 0.14 (SD = 0.67, 95% LA = −1.46 to 1.17) for sitting, −0.13 (SD = 0.80, 95% LA = −1.70 to 1.43) for speech, −0.16 (SD = 0.51, 95% LA = −1.16 to 0.84) for nose‐finger test, −0.07 (SD = 0.49, 95% LA = −1.03 to 0.88) for finger chase, −0.03 (SD = 0.64, 95% LA = −1.27 to 1.21) for fast alternating hand movements, and 0.00 (SD = 0.45, 95% LA = −0.87 to 0.87) for heel‐shin slide. ICC (consensus rating and live rating by different raters) was generally high with 0.98 for total SARA and 0.82 to 0.98 for single item analysis (0.98 for gait, 0.97 for stance, 0.85 for sitting, 0.90 for speech, 0.82 for nose‐finger test, 0.87 for finger chase, 0.88 for fast alternating hand movements, and 0.93 for heel shin slide).

## Discussion

Video recordings of neurological examinations are a frequently used means of documenting movement disorders. In this study, we compared live and remote video rating of identical SARA examinations. This study confirms that SARA ratings based on videos that were recorded under standardized conditions are equivalent to live ratings by the examiner. The variability between live and video ratings lies within the range of inter‐rater variability of SARA.^8^

Although all calculated levels of agreement from Bland–Altman analysis were excellent, there were slight differences between biases from the single item analysis. Explanations for this cannot be derived from the current study and further research is needed here. Potential causes may involve a mixture of more objective (numeric deviations from an ideal, eg, performance time for repetition of 10 fast alternating hand movements or standing in a defined a position, and number of deviations from the tibia during heel‐shin slide) and subjective criteria (eg, intelligibility during free speech). A detailed description of the score and item features can be found in the SARA instructions and the SARA training tool (https://www.ern-rnd.eu/sara-training-tool-by-dzne/).^1^ In fact, more objective items showed the greatest level of agreement between live and remote video raters in Bland–Altman analysis, with the smallest bias for items 2 (stance) and 8 (heel‐shin slide), followed by item 7 (fast alternating hand movements). Items 1 (gait) and 3 (sitting) performed worse than item 2 (stance). This is surprising, because correlation between these items is usually high and they are often summarized as trunk items. These items have a less clear and sometimes more subjective distinction between the score categories, that may explain the greater bias compared to the stance item. Inter‐rater variability may also contribute to these differences. The greatest bias was observed for item 5 (nose‐finger test). Although score categories are defined by objective criteria in this item (amplitude of tremor in cm), the exact distinction, for example between a 4‐ and 5‐cm amplitude of intention‐tremor, is often hard to detect during live examination. This study used a standardized protocol for video capture. However, we cannot exclude that small changes of perspective may have influenced video ratings. In addition, distinction between tremor, which is specifically rated in item 6, and other movement abnormalities observed in the nose‐finger test may be challenging.

In general, videos carry more information than written documentation. This enables remote physicians to build their own opinion based on videos, and examination results at the given time‐point become reproducible. Further advantages include a higher resolution in time through slow‐motion function and the possibility to replay in case of missed details. Limitations of video recordings compared to live examinations can arise from the loss of three‐dimensionality and selection of camera perspective.^9^ Nevertheless, video recordings are generally considered an adequate tool for training and educational purposes and are also used in clinical trials with centralized ratings. In this study, we used a standardized set‐up to ensure optimal video quality.

Although the video‐raters are affiliated with two different study sites, all recordings were obtained monocentric. Live ratings from investigators from different study sites may have shown greater variability, which is a limitation to this study. The use of a standardized set‐up for all SARA recordings is highly important to capture all necessary information for video raters. Results may, therefore, not be generalized to all videos from neurologic examinations. Another limitation arises through the use of consensus ratings. Comparison to live ratings from different raters introduces inter‐rater variability additionally to the two different modes of rating. Inter‐rater variability based on video ratings remains to be investigated in a follow‐up study.

Unlike many other scales, SARA involves very few rater‐patient interactions. The proof of equivalence of recorded and live SARA examinations, therefore, provides preliminary evidence that investigations may be performed without presence of an examiner, although this may not be feasible for all SARA items because of practicability (eg, necessity of a moving target for the finger‐chase) and safety concerns (eg, tandem walk and stance). The studied patient population was not fully representative of the whole spectrum of ataxia severity covered by SARA, as patients with high SARA scores were underrepresented. Consequently, conclusions may not apply to patients with very high SARA scores. A possible next step could be to move the examination, or at least parts of it, to the patient's home. Demonstration of feasibility of SARA home recordings and comparability of self‐performed SARA scores at home to examinations by a physician in the clinic have already been conducted in smaller cohorts.^6^, ^7^ Moving an examination, or at least parts of it, to patient's home is not only well accepted, but it is also closer to patients’ daily life surroundings, easier to integrate in their daily life, and allows higher frequent sampling, which might in turn, allow a higher signal to noise ratio.^6^, ^7^, ^10^ In addition, from the patient's perspective, home recordings reduce the burden of travel and risk of infection. From the examiner's perspective, home recordings are resource efficient, and therefore, allow for a higher frequency of examinations, for example in the context of treatment surveillance and/or symptom fluctuation.

Reproducibility of research results via video recordings represents a step toward automation of assessments through motion tracking and voice analysis. These developments may further improve efficiency especially in the context of immense video data generated through multicentric, longitudinal clinical trials.

## Author Roles

(1) Research project: A. Conception, B. Organization, C. Execution; (2) Statistical Analysis: A. Design, B. Execution, C. Review and Critique; (3) Manuscript Preparation: A. Writing of the First Draft, B. Review and Critique.

A.T.A.: 1A, 1B, 1C, 2A, 2B, 3B.

J.F.: 1C, 3B.

D.Ö.: 1C, 3B.

O.K.: 1C, 3B.

M.S.: 1C, 3B.

T.A.: 1A, 3B.

T.K.: 1A, 1B, 1C, 3B.

M.G.E.: 1A, 1B, 1C, 2A, 2C, 3A.

## Disclosures

**Ethical Compliance Statement**: This study was approved by the ethics committee of the University hospital Bonn (IRB: 212/19). Written and informed consent was obtained by all patients before participation. We confirm that we have read the Journal's position on issues involved in ethical publication and affirm that this work is consistent with those guidelines.

**Funding Sources and Conflicts of Interest**: This work was supported, in part, by the EJP RD WP20 Innovation Statistics consortium “EVIDENCE‐RND” under the EJP RD Grant Agreement (no. 825,575). The authors declare that there are no conflicts of interest relevant to this work.

**Financial Disclosures for the Previous 12 Months**: T.K. is receiving research support from the Bundesministerium für Bildung und Forschung (BMBF), the National Institutes of Health (NIH), and Servier. Within the last 24 months, he has received consulting fees from Biogen, UCB. and Vico Therapeutics. T.A. received grants from the NIH (NS104326), Biogen, and Biohaven. M.S. has received consultancy honoraria from Janssen, Ionis, Orphazyme, Servier, Reata, GenOrph, and AviadoBio, all unrelated to the present manuscript. The authors whose names are listed above certify that they have NO affiliations with or involvement in any organization or entity with any financial interest or non‐financial interest in the subject matter or materials discussed in this manuscript.

## Supporting information


**Figure S1.** SARA video protocol. All items were recorded according to a standardized protocol. (A) Set‐up instructions SARA Items 1 and 2. (B) Set‐up instructions for SARA Items 3–7. (C) Setup instructions for SARA Item 8.

**Figure S2.** Histogram of live (gray) and remote (black) ratings.

**Figure S3.** Bland–Altman‐analysis of single SARA item.
