# Supplementary figures and images for: Comparison of Live and Remote Video Ratings of the Scale for Assessment and Rating of Ataxia
Source: Mov Disord Clin Pract. 2023 Aug 7;10(9):1404–7. doi: 10.1002/mdc3.13843 (PMC10525045; doi:10.1002/mdc3.13843)

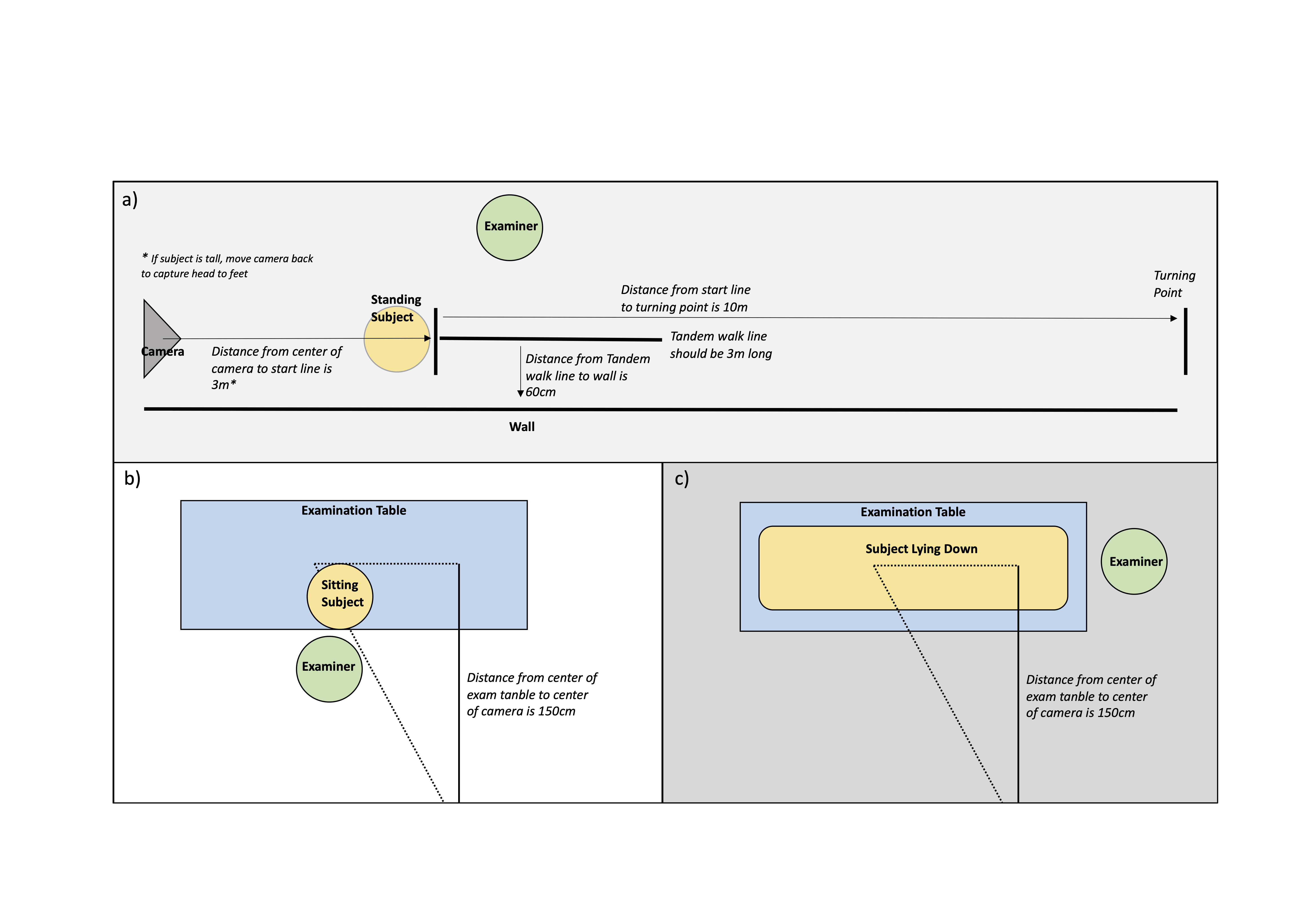

Supplement: Supplementary file 1 — Figure S1. SARA video protocol. All items were recorded according to a standardized protocol. (A) Set‐up instructions SARA Items 1 and 2. (B) Set‐up instructions for SARA Items 3–7. (C) Setup instructions for SARA Item 8. [file MDC3-10-1404-s001.png]

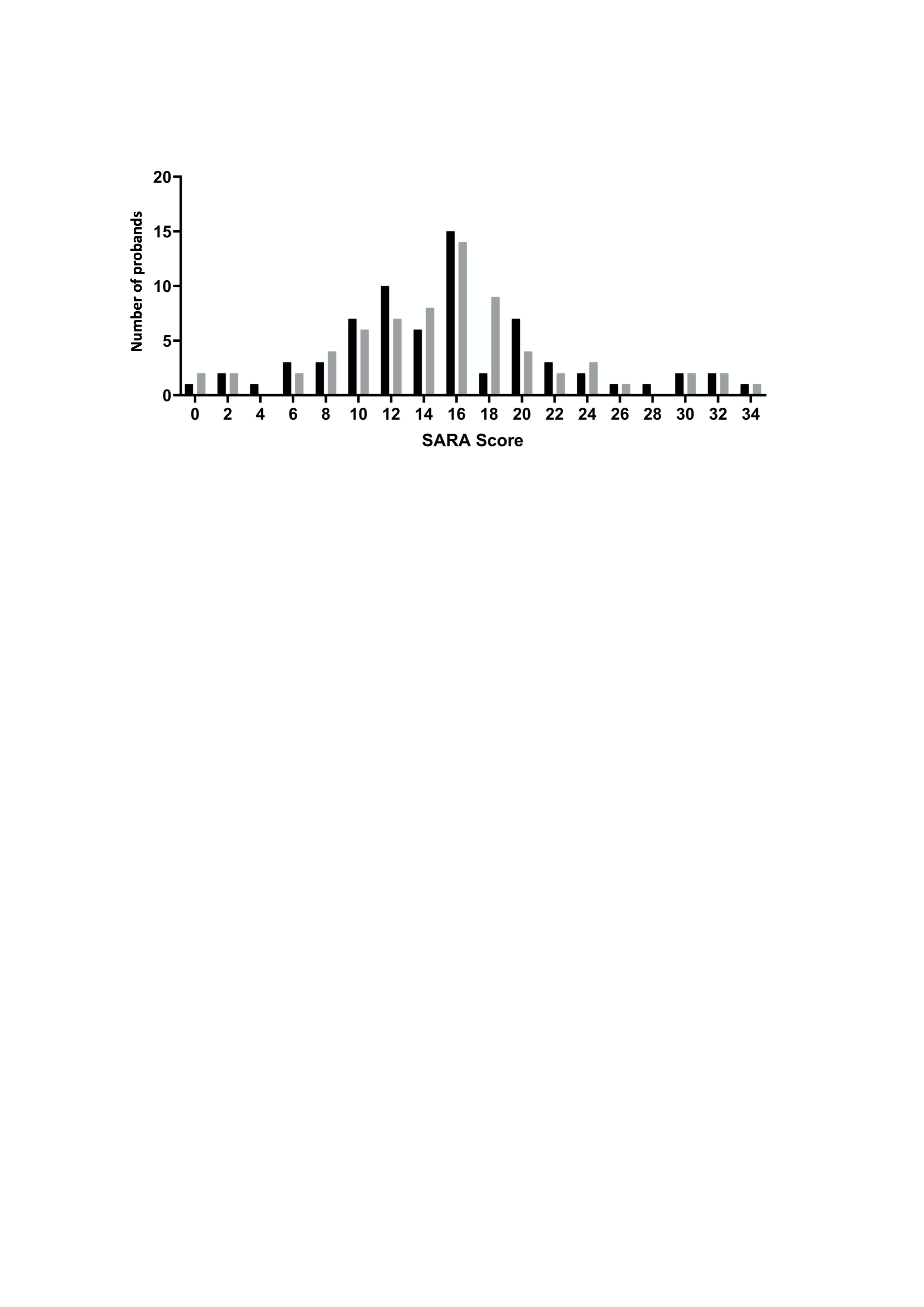

Supplement: Supplementary file 2 — Figure S2. Histogram of live (gray) and remote (black) ratings. [file MDC3-10-1404-s002.png]

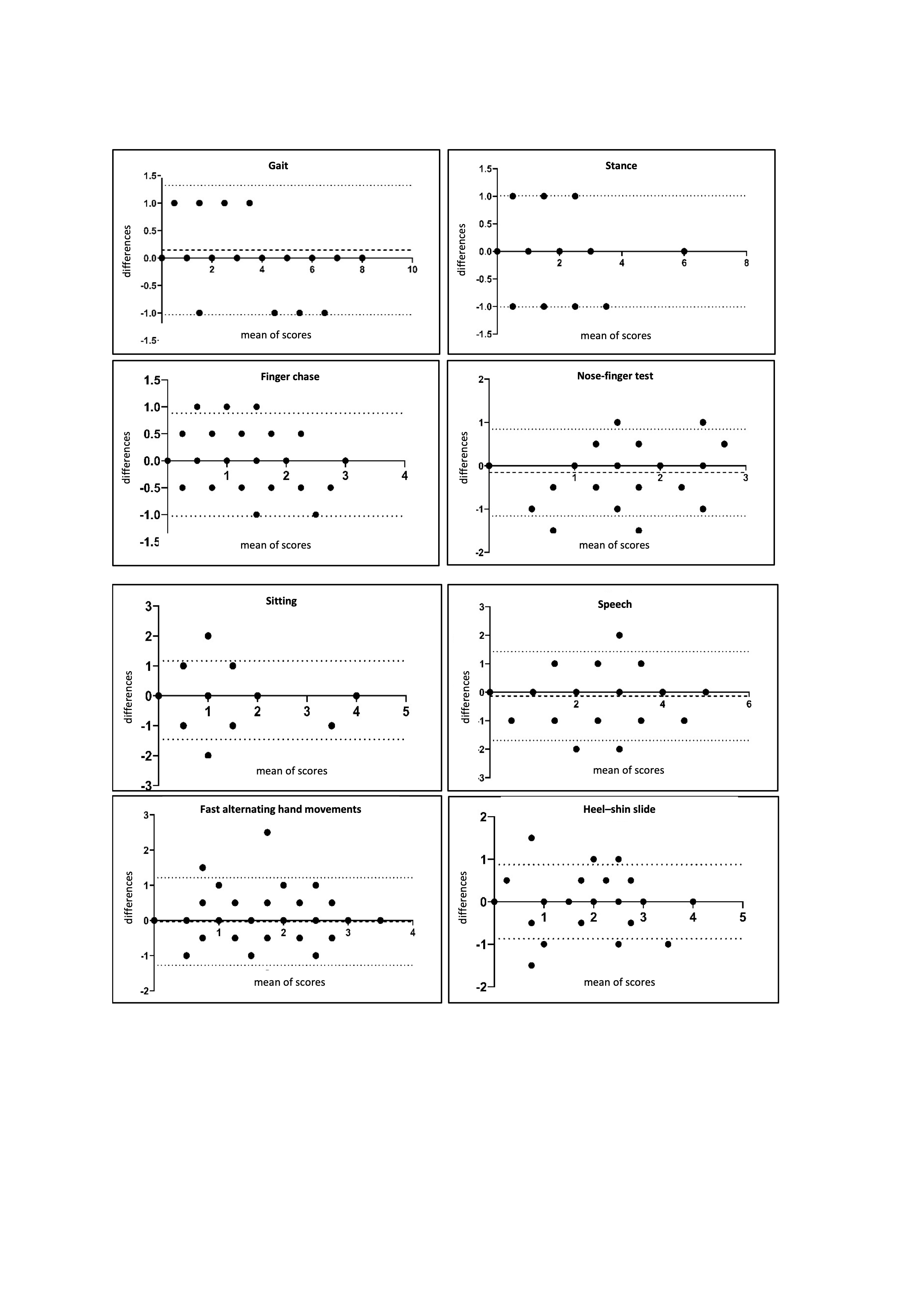

Supplement: Supplementary file 3 — Figure S3. Bland–Altman‐analysis of single SARA item. [file MDC3-10-1404-s003.png]
